# Retraction to “MiR-223-3p regulates cell viability, migration, invasion, and apoptosis of non-small cell lung cancer cells by targeting RHOB”

**DOI:** 10.1515/biol-2023-0002

**Published:** 2024-08-26

**Authors:** Shufang Li, Yuping Feng, Yuxia Huang, Yu Liu, Yanxi Wang, Yan Liang, Hui Zeng, Hong Qu, Ling Wei

**Affiliations:** Department of Respiratory Medicine, Lanzhou University Second Hospital, No. 82, Cuiyingmen, Linxia Rd, Chengguan District, 730030, Lanzhou, China; Department of Emergency, Gansu Provincial Third People’s Hospital, 730000, Lanzhou, China

The publisher would like to inform that the article “Li S, Feng Y, Huang Y, Liu Y, Wang Y, Liang Y, Zeng H, Qu H, Wei L. MiR-223-3p regulates cell viability, migration, invasion, and apoptosis of non-small cell lung cancer cells by targeting RHOB. Open Life Sciences. 2020;15(1): 389–399. https://doi.org/10.1515/biol-2020-0040” has been retracted.

This article has been retracted by agreement between the authors, the journal Editor-in-Chief, Professor Thomas Litman, and Walter De Gruyter GMBH. The retraction has been agreed at the request of the corresponding author Dr. Yuxia Huang, due to a previously undisclosed issue with ethical approval for human studies. The retraction has been approved by all co-authors.

